# Melatonin exerts immunoregulatory effects by balancing peripheral effector and regulatory T helper cells in myasthenia gravis

**DOI:** 10.18632/aging.103785

**Published:** 2020-11-02

**Authors:** Ting Chang, Chunxiao Niu, Chao Sun, Ying Ma, Rongjing Guo, Zhe Ruan, Yanwu Gao, Xiaodan Lu, Huanhuan Li, Ye Lin, Jiaji Lin, Zhuyi Li

**Affiliations:** 1 Department of Neurology, Tangdu Hospital, The Air Force Medical University, Xi’an, Shaanxi Province, P.R. China; 2 Department of Immunology, The Air Force Medical University, Xi’an, Shaanxi Province, P.R. China; 3 Department of Neurology, Chinese PLA General Hospital, Beijing, P.R. China; 4 Medical Corp in Unit 93246 of PLA, Changchun, Jilin Province, P.R. China

**Keywords:** myasthenia gravis, melatonin, melatonin receptor, Th cell. regulatory T cells, FoxP3

## Abstract

Myasthenia gravis (MG) is a prototypic organ-specific autoimmune disorder that, in most cases, is mainly mediated by antibodies against the acetylcholine receptor. Evidence implicates CD4^+^ T helper (Th) cells in the development of MG, whereas regulatory T cells (Tregs) are associated with disease resolution. Melatonin has important immunoregulatory effects in many T cell-mediated autoimmune diseases. However, there are few studies on the role of melatonin in MG. In the present study, we investigated serum melatonin levels and melatonin receptor expression in MG patients and healthy controls (HCs). We also evaluated the impact of melatonin administration on peripheral CD4^+^ Th cells and related cytokine production. Serum melatonin levels were lower in MG patients than in HCs, and MT1 expression was lower in PBMCs from MG patients than in those from HCs. Administration of melatonin significantly decreased Th1 and Th17 cell responses and proinflammatory cytokine production. Further investigation *in vitro* revealed that melatonin administration increased FoxP3 and IL-10 expression in CD4^+^ T cells from MG patients and enhanced the suppressive function of Tregs. These findings indicate that melatonin exerts immunoregulatory activity in MG by balancing effector and regulatory Th cell populations as well as by suppressing proinflammatory cytokine production.

## INTRODUCTION

Myasthenia gravis (MG) is a prototypic organ-specific autoimmune disorder generally mediated by antibodies against the acetylcholine receptor (AChR) in skeletal muscle at the neuromuscular junction (NMJ); these antibodies damage NMJs and impair neuromuscular transmission. In addition to humoral immunity, cellular immunity plays a crucial role in the pathogenesis of MG. In particular, the role of CD4^+^ T helper (Th) cells in the development of MG in humans and experimental models has become increasingly evident [1].

Melatonin, a major secretory hormone synthesized and secreted by the pineal gland in response to darkness, primarily regulates circadian rhythm. Melatonin also has many other physiological effects, including antioxidant and immunomodulatory effects. As an immune modulator, melatonin exhibits both pro- and anti-inflammatory activities [2]. Proinflammatory actions have been documented by many preclinical studies on isolated cells or leukocyte-derived cell lines, enhancing defense capacity against pathogens [3–5]. Such pro-inflammatory action has also been observed in human arthritis [6]. Melatonin often exerts physiological functions by interacting with its membrane receptors (MT1 and MT2) and nuclear receptors. Moreover, some well-designed investigations have demonstrated that T cells express membrane and nuclear receptors for melatonin [7]. Indeed, melatonin influences biological processes in T cells, including development, activation, differentiation, and even memory [7]. Recently, a number of *in vivo* and *in vitro* studies have revealed that melatonin plays a fundamental role in neuroimmunomodulation [7–9].

The regulation of T cell-mediated immune responses by melatonin has prompted immunologists to study the role of melatonin in various inflammatory and autoimmune diseases [6, 10–14]. One such disease, multiple sclerosis (MS), is a chronic neuroinflammatory disease of the central nervous system. Melatonin has been shown to alleviate the symptoms of experimental autoimmune encephalomyelitis (EAE) and a preclinical MS model and to decrease the incidence of EAE by suppressing peripheral and central Th1/Th17 responses while elevating Treg cell responses [15, 16]. Additionally, melatonin has been found to increase the ratio of anti-inflammatory cytokine/Th1 in relapsing-remitting MS patients and to promote the development of a protective cytokine microenvironment [17]. Systemic lupus erythematosus (SLE) is a typical autoimmune disease characterized by the production of antinuclear autoantibodies. Some studies have suggested that melatonin exerts beneficial effects in a lupus mouse model by regulating cytokine disturbances [14, 18, 19]. Furthermore, melatonin increases the number of FoxP3-expressing Tregs and attenuates BAFF overexpression in cells from SLE patients.

Regardless, to our knowledge, there have been few studies on the role of melatonin in MG in humans or experimental models. With the aim of investigating the potential effect of melatonin on T cells in autoimmune MG, we first detected circulating melatonin levels and expression of membrane and nuclear receptors for melatonin in peripheral blood mononuclear cells (PBMCs) and CD4^+^ Th cells of MG patients. Next, we evaluated the impact of melatonin administration on peripheral CD4^+^ Th cells and production of related cytokines. The findings will open new avenues of research on melatonin as a therapeutic agent for MG.

## RESULTS

### Serum melatonin and melatonin receptor (MT1) expression in PBMCs of MG patients

**Figure 1:**
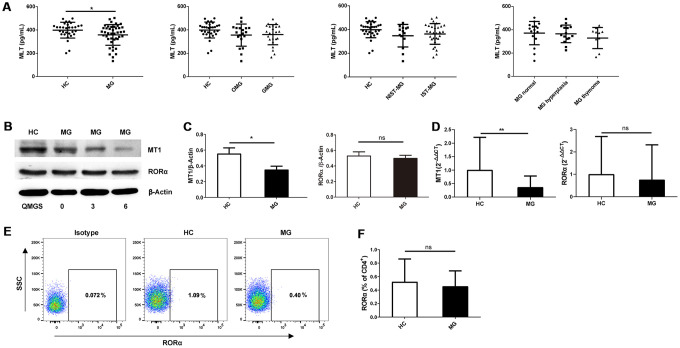
**Serum melatonin levels and melatonin receptor expression in MG patients and HCs.** (**A**) Serum melatonin levels among different MG subgroups (HC, n=31; MG, n=43; OMG, n=19; GMG, n=24; NIST-MG, n=14; IST-MG, n=29; MG normal, n=17; MG hyperplasia, n=15; MG thymoma, n=11). (**B**) Western blot analysis of MT1 and RORα expression in PBMCs from HCs and MG patients with different QMGS. The results are representative of three independent experiments. (**C**) Statistical analysis of MT1/β-Actin and RORα/β-Actin in different MG patients and HCs (HC, n=8; MG, n=13). (**D**) Relative expression of MT1 and RORα (HC, n=11; MG, n=35). The results are representative of three independent experiments. (**E**) FCM analysis of RORα expression in CD4^+^ T cells from HCs and MG patients. (**F**) Statistical analysis of RORα expression in CD4^+^ T cells (HC, n=6; MG, n=14). The data are presented as the mean ± SD. * *P* ≤ 0.05, ** *P*≤ 0.01 and ns=no significance.

Serum melatonin levels were investigated in various subgroups. The results showed that serum melatonin levels were lower in MG patients than in healthy controls (HCs); however, the difference was not observed in different MG subgroups (Figure 1A). Expression of melatonin-related receptors was assessed by Western blot and quantitative RT-PCR assays. According to Western blot analysis, MT1 expression was lower in PBMCs from MG patients than in those from HCs, whereas no difference in retinoic acid-related orphan receptor (ROR)α expression was observed between these groups (Figure 1B, 1C). RT-PCR analysis showed that MT1 expression was significantly lower in PBMCs of MG patients than in those from HCs, with no statistically significant difference in RORα expression between MG patients and HCs (Figure 1D). Moreover, RORα expression was detected in peripheral CD4^+^ Th cells and was not significantly lower in cells from MG patients than in those from HCs (Figure 1E, 1F). Further correlation analysis indicated no correlation between melatonin receptor expression or serum melatonin levels and quantitative MG score (QMGS) (Table 1). Based on Spearman’s correlation analysis, serum melatonin levels did not correlate with the quantitative MG score (QMGS) (r = 0.216, *P*=0.311). Similarly, there was no association between melatonin receptor MT1 and RORα expression in PBMCs and QMGS (r =-0.232, *P*=0.658; r =0.166, *P*=0.753, respectively) or between RORα expression in CD4^+^ Th cells and QMGS (r = 0.295, *P*=0.306).

**Table 1:** Nonparametric correlation analysis between serum melatonin levels or melatonin receptor expression and QMGS (MG patients only).

	**MG patients**	
	***r***	***P*-value**
Serum melatonin level (pg/ml)	0.216	0.311
RORα expression in CD4^+^ T cells	0.295	0.306
MT1 expression in PBMCs	-0.232	0.658
RORα expression in PBMCs	0.166	0.753

### Effect of melatonin treatment on Th1 and Th17 cell responses

**Figure 2:**
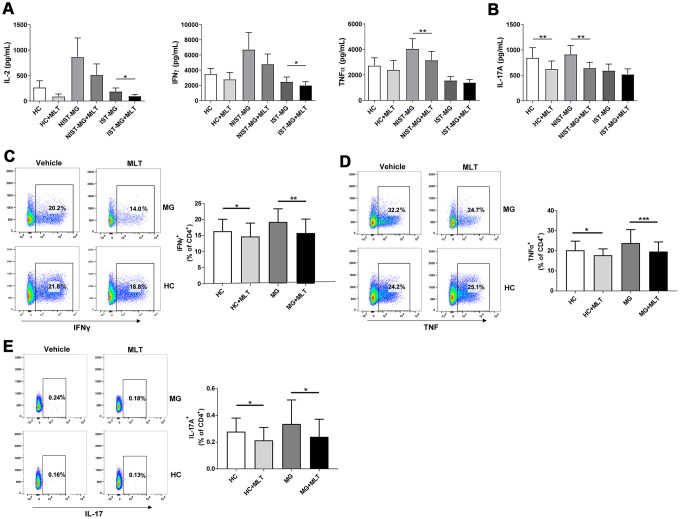
**Melatonin decreased Th1 and Th17 cell responses.** (**A**) Alterations in the production of signature Th1 cytokines, including IL-2, IFNγ and TNFα, after melatonin treatment in different MG subgroups. (**B**) Levels of IL-17A in response to melatonin treatment (HC, n=10; NIST-MG, n=13; IST-MG, n=15). (**C**) The frequency of CD4^+^ T cells expressing either IFNγ or (**D**) TNFα in melatonin-treated PBMCs from MG patients and HCs. (**E**) The frequency of CD4^+^ T cells expressing IL-17A in melatonin-treated PBMCs (HC, n=10; MG, n=10). The data are presented as the mean ± SD. * *P* ≤ 0.05, ** *P* ≤ 0.01, *** *P* ≤ 0.001.

A previous study demonstrated that both Th1 and Th17 cells are involved in the pathogenesis of MG. In the present study, we found higher levels of Th1 cell hallmark cytokines (IL-2, IFNγ, and TNFα) in the nonimmunosuppressive therapy-MG (NIST-MG) group than in the HC and immunosuppressive therapy-MG (IST-MG) groups. Melatonin significantly decreased the levels of Th1 cell hallmark cytokines in the culture supernatants of PBMCs from MG patients (Figure 2A). In addition, levels of IL-17A, a Th17 cell cytokine, were decreased in both HCs and NIST-MG patients after melatonin treatment (Figure 2B). This finding suggests that melatonin can suppress proinflammatory cytokine production; however, it is difficult to assign the production of cytokines to a particular T cell subset with such an approach using PBMCs as the cell source. Therefore, we subsequently investigated the frequency of different T cell subgroups after melatonin treatment and found that the frequency of CD4^+^ T cells expressing either IFNγ or TNFα was lower in melatonin-treated PBMCs than in vehicle-treated PBMCs from either MG patients or HCs (Figure 2C, 2D). Additionally, the frequency of CD4^+^ T cells expressing IL-17A in PBMCs decreased after melatonin treatment (Figure 2E). Decreased proinflammatory cytokine levels and flow cytometry (FCM) results may provide some indication that melatonin can decrease Th1 and Th17 cell responses.

### *In vitro* effect of melatonin administration on proinflammatory cytokines

**Figure 3:**
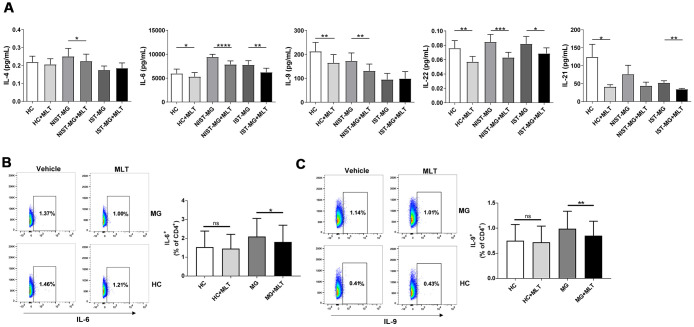
***In vitro* melatonin administration attenuated the production of other proinflammatory cytokines.** (**A**) IL-4, IL-6, IL-9, IL-22 and IL-21 levels in PHA-stimulated PBMC culture supernatant after melatonin administration in different MG subgroups (HC, n=10; NIST-MG, n=13; IST-MG, n=15). (**B**) FCM analysis of the frequency of CD4^+^ T cells producing IL-6 and (**C**) IL-9 and representative plots (HC, n=10; MG, n=10). The data are presented as the mean ± SD. * *P* ≤ 0.05, ** *P* ≤ 0.01, *** *P* ≤ 0.001, **** *P* ≤ 0.0001.

In the NIST-MG group, levels of IL-4, a key Th2 cell cytokine, decreased slightly after melatonin treatment. Moreover, IL-6 levels were markedly reduced after melatonin treatment in PBMCs from both HCs and MG patients, with a particular decrease for the NIST-MG patient group. Moreover, the levels of IL-9, a representative Th9 cell cytokine, decreased in PBMCs from the HC and MG patient groups, especially in the NIST-MG group, after melatonin treatment. Levels of IL-22, a representative Th22 cell cytokine, were markedly lower after melatonin treatment in PBMCs from HCs and MG patients, especially in the NIST-MG group. IL-21 levels were also markedly decreased by melatonin treatment in HCs and IST-MG patients (Figure 3A). Next, we performed intracellular cytokine staining for two representative cytokines. The frequencies of CD4^+^ T cells expressing IL-6 (Figure 3B) or IL-9 (Figure 3C) were lower in melatonin-treated PBMCs from MG patients than in vehicle-treated cells.

### Effect of melatonin on CD4^+^ T cell proliferation in HCs and MG patients

**Figure 4:**
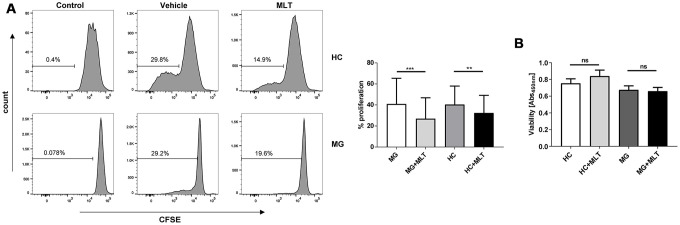
**Melatonin significantly decreased the proliferation of CD4^+^ T cells isolated from either MG patients or HCs but did not alter cell viability.** (**A**) CFSE assay of PHA-stimulated proliferation of melatonin- or vehicle-treated CD4^+^ T cells. (**B**) Viability of melatonin-stimulated or unstimulated CD4^+^ T cells (HC, n=10; MG, n=10). The data are presented as the mean ± SD. ** *P* ≤ 0.01, *** *P* ≤ 0.001 and ns=no significance.

To evaluate whether the effect of melatonin on CD4^+^ T cells involves a change in cell viability, we analyzed potential differences in the proliferation and viability of melatonin- or vehicle-treated CD4^+^ T cells from both HCs and MG patients. Melatonin significantly inhibited the proliferation of CD4^+^ T cells from both groups (Figure 4A), though there was no difference in CD4^+^ T cell viability between the HC and MG groups (Figure 4B).

### Effect of melatonin on FoxP3 and IL-10 expression and function of Tregs *ex vivo*

**Figure 5:**
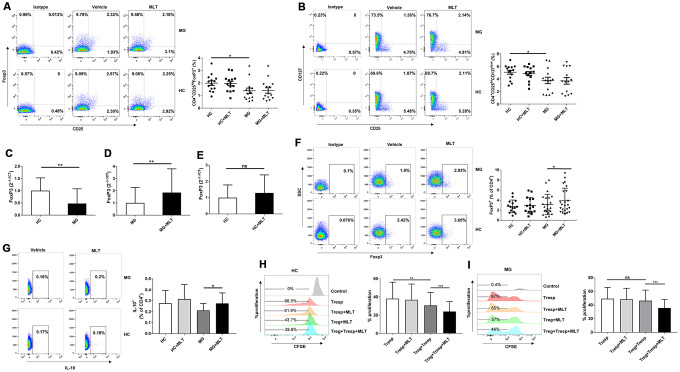
**Melatonin increased FoxP3 and IL-10 expression and enhanced the suppressive function of Tregs *ex vivo.*** (**A**) Frequency of CD4^+^CD25^hi^FoxP3^+^ Tregs after melatonin stimulation (HC, n=14; MG, n=14). (**B**) Frequency of CD4^+^CD25^hi^CD127^low^ Tregs (HC, n=14; MG, n=14). (**C**) Relative expression of FoxP3 in PBMCs from MG patients and HCs (HC, n=14; MG, n=38). The results are representative of three independent experiments. Relative expression of FoxP3 after melatonin treatment in MG patients (**D**) and HCs (**E**). (**F**) FoxP3 expression in CD4^+^ T cells of MG patients and HCs after melatonin treatment (HC, n=15; MG, n=23). (**G**) IL-10 expression in CD4^+^ T cells of MG patients and HCs after melatonin treatment (HC, n=10; MG, n=10). CD4^+^CD25^hi^CD127^low^ Tregs and CD4^+^CD25^-^ Tresps were sorted. Tresps were labeled with CFSE and cocultured with Tregs with or without melatonin in the presence of PHA. Four days later, the percentage of Tresps was determined based on CFSE dilution and FCM. (**H**) CD4^+^ T cells enriched from HCs. (**I**) CD4^+^ T cells enriched from MG patients (HC, n=10; MG, n=10). The data represent the mean ± SD. * *P* ≤ 0.05, ** *P* ≤ 0.01, *** *P* ≤ 0.001 and ns=no significance.

As depicted in Figure 5A, 5B, the frequency of Tregs, either CD4^+^CD25^hi^FoxP3^+^ or CD4^+^CD25^hi^CD127^low^ cells, was lower in MG patients than in HCs. The number of Tregs in HCs and MG patients remained unchanged after melatonin treatment. However, RT-PCR demonstrated that FoxP3 expression was significantly downregulated in MG patients compared with HCs, indicating a functional defect in Tregs from the former group (Figure 5C). Furthermore, FoxP3 expression was upregulated in MG patients in response to melatonin treatment, and this response was not observed in HCs (Figure 5D, 5E). FCM analysis also revealed that melatonin increased FoxP3 expression in CD4^+^ T cells from MG patients but had little effect on those from HCs (Figure 5F). Expression of IL-10 was also increased in MG patients treated with melatonin treatment (Figure 5G). Tregs from HCs significantly inhibited the proliferation of Tresps (Figure 5H), but those from MG patients could not effectively inhibit Tresp proliferation, indicating a functional defect of Tregs in MG patients (Figure 5I). The suppressive effect of Tregs on phytohemagglutinin (PHA)-stimulated Tresp proliferation was stronger in the presence than in the absence of melatonin in both HCs and MG patients (Figure 5H, 5I).

## DISCUSSION

Melatonin concentrations have been investigated in several autoimmune diseases, with variable results. Robeva et al. reported that women with SLE had decreased daily melatonin levels and that daily melatonin concentrations were inversely related to disease severity [12]. In contrast, Wang et al. found that plasma melatonin levels did not differ between SLE patients and controls, with no significant correlation between melatonin levels and major clinical or laboratory features in SLE patients [20]. We first observed lower serum melatonin levels in MG patients than in HCs, though we did not find any difference in melatonin levels among MG subtypes. Further correlation studies suggested that melatonin levels may not be closely related to disease severity or immunotherapy. Several well-designed investigations have demonstrated the importance of both membrane and nuclear molecules for melatonin-mediated activation of T cell function. For example, treatment of mice with a high-affinity antagonist of MT1/MT2 inhibits the ability of melatonin to stimulate splenic lymphocyte proliferation in response to the T cell mitogen concanavalin A [21]. In our study, expression of the membrane melatonin receptor MT1, but not RORα, was significantly lower in PBMCs from MG patients than in those from HCs at both transcriptional and protein levels. This observation suggests that decreased MT1 expression in MG patients may influence the modulatory function of melatonin in T cells; the mechanisms, however, have yet to be defined.

Pathogenic anti-AChR antibodies play a crucial role in the development of MG and experimental autoimmune MG (EAMG), though their synthesis, class switching and somatic hypermutation require help from autoreactive CD4^+^ T cells. A previous study showed the presence of AChR-autoreactive T cells in the blood and thymus of MG patients. These AChR-autoreactive T cells exert proinflammatory and pathogenic activity by elevating IL-17, IFN-γ, and GM-CSF expression and diminishing IL-10 production [22]. According to our study, melatonin administration significantly decreased the levels of these proinflammatory cytokines and increased expression of IL-10 in MG patients, indicating that melatonin may suppress the AChR-autoreactive T cell response.

Disequilibrium of Th cell populations plays an important role in the development of EAMG and the pathogenesis of MG [1, 23]. The pathogenic role of Th1 cells and their cytokines in MG and EAMG has been described in several well-designed studies. Th17 cells, defined as an effector Th cell subgroup in the past decade, have critical functions in many autoimmune and inflammatory diseases by secreting the major cytokine IL-17A. The Th17 cell population in PBMCs and serum IL-17A concentrations are significantly increased in MG patients and correlate with anti-AChR antibody titer and disease severity [24–26]. Moreover, Th17/Treg cell imbalance has been implicated in the development of MG [27], and a previous study revealed that Th17 cells and IL-17 contribute to EAMG development through the loss of B cell tolerance [28]. In the present study, treatment with melatonin led to reductions in Th17 cell number and IL-17A and IL-21 production, suggesting that melatonin decreases the Th17 cell response. The cytokines IL-6 and TGF-β reportedly promote the differentiation of Th cells into mature Th17 cells [29]. Our study found that melatonin decreased IL-6 expression, implicating that melatonin has the potential to suppress Th17 differentiation.

Studies over the past decade have highlighted the importance of IL-21 in humoral immunity in humans. IL-21 has been reported to exert all of its functions on human B cells; for example, IL-21 is a potent growth and differentiation factor for human B cells. It efficiently promotes class switching and production of large quantities of immunoglobulins in activated B cells [30, 31]. In addition, IL-21 can induce expression of B cell lymphoma 6 (Bcl-6) in human naïve B cells, suggesting that IL-21 has a role in establishing the germinal center. Ectopic germinal center formation plays a crucial role in the pathogenesis of MG, and our previous study demonstrated that interactions between Tfh and B cells in the thymus of patients with MG can promote ectopic germinal center formation and autoantibody secretion through the effector molecule IL-21. We also found high levels of the cytokine IL-21 in both hyperplastic thymus samples and peripheral blood from patients with MG, suggesting that IL-21 is involved in the pathogenesis of MG [32]. In the current study, melatonin administration significantly decreased IL-21 production in cultures of PBMCs from both MG patients and HCs. To our knowledge, this is the first report of the inhibitory effect of melatonin on IL-21 production. These findings suggest that melatonin influences ectopic germinal center formation and autoantibody production, and further studies are needed to determine whether melatonin affects antigen-specific B cells.

*In vitro* assessment of cytokine release and Th cell subsets revealed that melatonin significantly decreases proinflammatory cytokine production and suppresses effector Th cell responses. Moreover, we did not observe altered CD4^+^ T cell viability in cells from either HCs or MG patients after melatonin treatment, indicating that the inhibitory effect of melatonin on effector Th cell responses was not due to toxicity. Nonetheless, strong antiproliferative activity of melatonin against CD4^+^ Th cells from either MG patients or HCs was detected in the current study. The antiproliferative activity of melatonin has previously been described for various cells in several autoimmune diseases, such as T cells in nonobese diabetic (NOD) mice [33] and PHA-stimulated PBMCs in MS patients [17].

Tregs, which are distinct from other CD4^+^ Th cells, play a central role in maintaining peripheral tolerance and protecting against autoimmunity. FoxP3 is the key transcription factor for the suppressive activity of Tregs. Defective Treg function appears to be a common cause of human autoimmune diseases [34–37]. Indeed, a significant defect in Treg suppressive function has been described in MG [38] and in its animal model EAMG [27, 39]; a reduction in the frequency of CD4^+^CD25^+^FoxP3^+^ cells combined with decreased suppressive activity in EAMG has also been found [39], and a severe functional defect and decreased FoxP3 expression in thymic Tregs of MG patients have been reported [40]. In the current study, the number of Tregs remained unchanged after melatonin treatment, but FoxP3 expression was significantly upregulated in CD4^+^ T cells from MG patients. It is interesting that melatonin increased FoxP3 expression only in patient CD4^+^ Th cells but had little effect on HC cells. This result is partially in agreement with the results of a study by Medrano-Campillo et al., who observed that exogenous melatonin administration increased the frequency of FoxP3^+^ cells and FoxP3 mRNA levels in patients with SLE but did not affect the frequency of FoxP3^+^ cells in PBMCs from healthy individuals [13]. Regardless, how melatonin enhances FoxP3 expression in autoimmune patients remains unclear. Medrano-Campillo speculated that calcium/calmodulin-dependent kinase IV (CAMKIV) is a putative target of melatonin. CAMKIV levels are higher in *in vitro*-cultured T cells from untreated lupus patients than in those from control patients, and silencing CAMKIV gene expression increases FoxP3 production after TGF-β stimulation. Thus, the mechanism by which melatonin enhances FoxP3 expression needs to be further studied. Although melatonin cannot change the number of Tregs, the suppressive function of Tregs was enhanced in response to melatonin. This suppressive function was further demonstrated by the stronger ability of melatonin-treated Tregs than vehicle-treated Tregs to suppress PHA-stimulated proliferation in Tresps from MG patients and HCs. Combined with the decreased Th17 cell response, we presume that melatonin has the potential to restore the Th17/Treg balance.

MG is a heterogeneous disease, and the differences in autoantibody patterns, HLA associations, thymic pathological changes, and clinical manifestations all point to differences in the pathogenesis of various MG subtypes. Therefore, we measured melatonin levels in different subgroups. However, in subsequent studies on melatonin receptor expression and intracellular cytokine measurement, we did not differentiate among these MG subgroups due to the small sample size. As immunotherapy can influence cytokine secretion, we grouped patients according to exposure to immunotherapy when analyzing cytokine levels in cultures of PBMCs. Furthermore, because immunotherapy-naïve patients better reflect pathology than do heterogeneously treated patients, we attempted to increase the number of such patients. Due to the rarity of MG, most patients had received immunotherapy at the time of recruitment, and only 14 immunotherapy naïve patients were included. In the future, our data must be verified with a larger cohort of patients encompassing more immunotherapy-naïve patients. Although our findings demonstrate the anti-inflammatory action of melatonin in MG, proinflammatory effects of melatonin exist, especially in rheumatoid arthritis [41, 42]. Thus, balancing the pro- and anti-inflammatory effects of melatonin is crucial, and the optimal dose should be investigated when exogenous melatonin is administered.

In summary, this study demonstrates, for the first time, that melatonin exerts important immunoregulatory activity in MG by balancing effector and regulatory CD4^+^ Th cells. Although the underlying regulatory mechanism remains to be completely elucidated, the results obtained in our study suggest that melatonin is a useful adjunctive therapy for MG.

## MATERIALS AND METHODS

### Patients and controls

In total, 43 MG patients with detailed clinical data were recruited from the Department of Neurology at Tang Du Hospital from December 2017 to December 2019. Thymic hyperplasia or thymoma was diagnosed based on chest radiological testing (either a computed tomography or magnetic resonance imaging scan). Thirty percent of patients underwent thymectomy (n=13), including 11 with thymoma and 2 with thymic hyperplasia. In one patient, other autoimmune diseases were present, including SLE, rheumatoid arthritis, Sjögren’s syndrome and autoimmune thyroid disease. Thirty-one HCs, 15 males and 16 females aged 29-67 years (mean, 40.9±11.1 years), were selected among volunteers in the local community. To rule out the effects of immunosuppressive drugs on cytokine secretion, we divided the MG patients into two groups: an NIST group of patients who did not receive immunotherapy, including corticosteroids, azathioprine, plasma exchange or intravenous immunoglobulin; an IST group of patients who were treated with the abovementioned immunosuppressive agents. MG is classified as either ocular or generalized based on the clinical status and affected muscles. Patients presenting only ocular symptoms such as diplopia and ptosis are designated having ocular MG (OMG); generalized MG (GMG) is characterized by muscle weakness involving the bulbar, neck, proximal extremity and even respiratory muscles. The patients were divided into three subgroups according to thymus abnormalities: MG with a normal thymus, MG with thymic hyperplasia and MG with thymoma. The characteristics of the included patients are shown in Table 2. The QMGS was used to evaluate disease severity, with a higher score indicating a more severe disease [43]. This study and the use of human PBMCs were approved by the local ethics committee and authorized by the National Key Research and Development Project-MG cohort study (2017YFC0907705). Both MG patients and HCs signed a consent form for the collection of data and blood samples. The study adhered to the Declaration of Helsinki guidelines for medical research. Peripheral blood samples were collected from MG patients and sex- and age-matched HCs in Vacutainer blood collection tubes (BD, USA) between 9:00 and 10:00 am and centrifuged. The serum was stored at -80°C until assayed. PBMCs were isolated by centrifugation (with Ficoll-Paque, GE Healthcare, USA) and cultured at 1×10^6^ cells/plate in RPMI 1640 medium (HyClone, USA) with 10% fetal calf serum.

**Table 2:** Characteristics of the study participants.

	**MG patients (n=43)**
**General characteristics**	
Sex, female/male (n)	20/23 (43)
Age at onset, years*	40.2±17.439
Age at onset <50 years (EOMG), n (%)	31 (72.1%)
Age at onset ≥50 years (LOMG), n (%)	12 (27.9%)
**Clinical characteristics**	
OMG, n (%)	19 (44.1%)
GMG, n (%)	24 (55.8%)
AChR antibody, n (%)	
Seropositive	30 (69.7%)
Seronegative	5 (11.6%)
NA	8 (18.6%)
Repetitive nerve stimulation, n (%)	
Normal	30 (69.7%)
Abnormal	12 (27.9%)
NA	1 (2.3%)
Thymic abnormality (chest radiology), n (%)	
Normal	17 (39.5%)
Thymic hyperplasia	15 (35%)
Thymoma	11 (25.5%)
Coexistence with other autoimmune diseases, n (%)	1 (0.03%)
**Medications**	
Immunosuppressive therapy, n (%)	14 (32.5%)
Nonimmunosuppressive therapy, n (%)	29 (67.4%)
Thymectomy, n (%)	13 (30%)

*mean ± SD; n: number; EOMG: early-onset myasthenia gravis; LOMG: late-onset myasthenia gravis; OMG: ocular myasthenia gravis; GMG: generalized myasthenia gravis; QMGS: quantitative myasthenia gravis score; NA: not available

### Cell culture and stimulation

PBMCs were cultured in the presence of 8 μg/ml PHA (Millipore, Sigma, USA) with 10^-4^ M melatonin (Millipore, Sigma, USA) or vehicle (0.02% dimethyl sulfoxide). After 48 hours of incubation at 37°C in a 5% CO_2_ humidified atmosphere, the cell culture supernatants were collected and stored at -80°C for cytokine assessment. Cells were collected for FCM analysis and RNA extraction.

### Serum melatonin measurement

Serum melatonin levels were measured with Human Melatonin ELISA Kits (Elabscience, China) according to the manufacturer's instructions. The enzyme-substrate reaction was terminated by the addition of stop solution, and the color change was measured spectrophotometrically at 450 nm. The concentration of human melatonin in the samples was determined by comparing the optical densities (ODs) of the samples to a standard curve.

### Flow cytometry

For ROR α detection, cells were surface stained with FITC-conjugated mouse anti-human CD4 antibodies (1:10, 550628, BD) for 30 minutes at 4°C and washed once with staining buffer. The cell pellets were resuspended by brief vertexing, and 1 ml of freshly prepared 1× Fix/Perm Buffer (BD, USA) working solution was added to each tube. The samples were incubated at 4°C for 50 minutes in the dark, after which the fixed and permeabilized cells were washed with 1× Perm/Wash Buffer (BD, USA). Intracellular proteins were stained by incubation at 4°C for 50 minutes with 100 μl of 1× Perm/Wash Buffer and PE-conjugated mouse anti-human RORα/NR1F1 (1:10, clone #784652, R&D Systems, USA). The cells were then washed twice, and the cell pellets were resuspended in 350 μl of FCM staining buffer. The cells were analyzed, and data were acquired using a flow cytometer (BD FACSAria II, USA). For Treg detection, cells were surface stained with FITC-conjugated mouse anti-human CD4 (1:10, 550628, BD), APC-conjugated mouse anti-human CD25 (1:5, 555434, BD), and PerCP-Cy5.5-conjugated mouse anti-human CD127 (1:20, 560551, BD); intracellular FoxP3 was stained with a PE-conjugated mouse anti-human FoxP3 (1:10, 560082, BD). All data were analyzed with FlowJo software.

### Cytokine staining and measurement

PBMCs were stimulated with phorbol myristate acetate (PMA), ionomycin and brefeldin A (423304, BioLegend) in the presence of 10^-4^ M melatonin (Millipore, Sigma) or vehicle (0.02% dimethyl sulfoxide). Six hours later, the activated cells were stained with FITC-conjugated mouse anti-human CD4 (550628, BD) at 4°C for 30 minutes. After surface staining, the cells were fixed and permeabilized for 1 hour at room temperature by BD Cytofix/Cytoperm buffer (BD, USA). Intracellular cytokine staining was performed with APC-conjugated mouse anti-human IL-10 (1:20, 501410, BioLegend), PE-conjugated mouse anti-human IL-17 (1:5, 560438, BD), PerCP-Cy5.5-conjugated rat anti-human IL-6 (1:20, 561118, BioLegend), PE-conjugated mouse anti-human TNFα (1:10, 559321, BD), APC-conjugated mouse anti-human IFNγ (1:10, 551385, BD), and PerCP-Cy5.5-conjugated mouse anti-human IL-9 (1:20, 507610, BioLegend). For cytokine measurement, the supernatant of unstimulated or melatonin-stimulated (48 hours) cell cultures was collected and assessed for nine cytokines (IFNγ, TNFα, IL-2, IL-4, IL-6, IL-9, IL-17A, IL-22, and IL-21) using Luminex technology (Human TH17 Magnetic Bead Panel, Merck, Germany).

### RNA isolation and quantitative RT-PCR

Total RNA was extracted with an RNAprep Pure Kit for cells/bacteria (Tiangen Biotech, China) according to the manufacturer’s instructions. A spectrophotometer (ScanDrop 200, Germany) was used to determine the RNA quantity and purity. The extracted RNA was translated into cDNA using a Transcriptor First Strand cDNA Synthesis Kit (Roche Diagnostics GmbH, Germany). Quantitative RT-PCR was performed in duplicate with a LightCycler 480 instrument (Roche Diagnostics GmbH, Germany). The primer sequences are provided in Table 3.

**Table 3:** RT-PCR primers for MT1, RORα and FoxP3.

**Gene**	**Sense 5→3**	**Antisense 5→3**	**Size (bp)**
MT1	CGTTGGTGCTGATGTCG	AGTTTGGGTTTGCGGTC	442
RORα	CTGACGAGGACAGGAGTAGG	GTGCGCAGACAGAGCTATTC	204
FoxP3	AGGAAAGGAGGATGGACGAA	GCAGGCAAGACAGTGGAAAC	123
GAPDH	TGCACCACCAACTGCTTAGC	GGCATGGACTGTGGTCATGAG	87

### Western blotting analysis

PBMCs were homogenized in RIPA lysis buffer (Servicebio, China). The protein concentration was determined using a BCA Protein Assay Kit (Servicebio, China). Protein (10 μg) was separated by 12% SDS-PAGE and transferred to a PVDF membrane, which was probed with a rabbit anti-melatonin receptor 1A antibody (1:100, Abcam, UK), a rabbit anti-RORα antibody (1:100, Abcam, UK) or a mouse anti-β-actin antibody (1:4000, Servicebio, China). A horseradish peroxidase-conjugated goat anti-rabbit IgG or a goat anti-mouse IgG secondary antibody (1:3000, Servicebio, China) was used as the secondary antibody. The specific protein bands were visualized with an ECL Plus Western blotting Detection Kit (Servicebio, China), and a ChemiDoc XRS system (Bio-Rad, USA) was used to image the bands. The images were analyzed with Gel-Pro Analyzer 4.0 software (Media Cybernetics, USA).

### Cell proliferation and viability assays

To evaluate proliferation, CD4^+^ T cells were purified from PBMCs using a CD4^+^ T Cell Isolation Kit (130096553, Miltenyi Biotec) and incubated with 5 μM CFSE (423801, BioLegend) in sterile PBS for 10 minutes at 37°C in the dark. The CFSE-labeled CD4^+^ T cells were washed with 10% FBS, cultured in 32-well plates and stimulated with PHA-L (00497793, Thermo) in the presence of 10^-4^ M melatonin (Millipore, Sigma) or vehicle (0.02% dimethyl sulfoxide) for 3 days. The inhibitory effect of melatonin on CD4^+^ T cell proliferation was assessed by CFSE dilution using FCM. Cell viability assays were performed after 48 hours of culture using a Cell Counting Kit (seven Sea Biotech, China) according to the manufacturer’s instructions.

### Treg suppression assay

CD4^+^CD25^hi^CD127^low/-^ Tregs were sorted from PBMCs from MG patients and HCs using a Treg Isolation Kit (130094775, Miltenyi Biotec) according to the manufacturer’s instructions. Responder T cells (Tresps), characterized as CD4^+^CD25^-^ cells, were similarly sorted from PBMCs of MG patients and HCs using a CD4^+^ T Cell Isolation Kit (130096553, Miltenyi Biotec) and CD25^-^ isolation (130092983, Miltenyi Biotec). Tresps were labeled with CFSE and cocultured with Tregs in the presence of PHA-L, 40 U/ml IL-2 (PeproTech, USA) and 10^-4^ M melatonin (Millipore Sigma) or vehicle (0.02% dimethyl sulfoxide) for 4 days. The cells were harvested, and Tresp proliferation was evaluated by CFSE dilution.

### Statistical analysis

Data are expressed as the mean ± SD after analysis with GraphPad Prism 5.0 software (GraphPad Software, San Diego, CA, USA). Differences between groups and paired samples were analyzed using *t*-tests. Nonparametric correlations between serum melatonin levels or melatonin receptor expression and QMGS were examined using Spearman’s rank correlation coefficient test with SPSS v24.0 software. *P*<0.05 was considered to indicate statistical significance.
